# Optimization of Carboxymethyl-Xyloglucan-Based Tramadol Matrix Tablets Using Simplex Centroid Mixture Design

**DOI:** 10.1155/2013/396468

**Published:** 2012-11-26

**Authors:** Ashwini R. Madgulkar, Mangesh R. Bhalekar, Rahul R. Padalkar, Mohseen Y. Shaikh

**Affiliations:** Department of Pharmaceutics, AISSMS College of Pharmacy, Kennedy Road, Pune 411001, Maharashtra, India

## Abstract

The aim was to determine the release-modifying effect of carboxymethyl xyloglucan for oral drug delivery. Sustained release matrix tablets of tramadol HCl were prepared by wet granulation method using carboxymethyl xyloglucan as matrix forming polymer. HPMC K100M was used in a small amount to control the burst effect which is most commonly seen with natural hydrophilic polymers. A simplex centroid design with three independent variables and two dependent variables was employed to systematically optimize drug release profile. Carboxymethyl xyloglucan (*X*_1_), HPMC K100M (*X*_2_), and dicalcium phosphate (*X*_3_) were taken as independent variables. The dependent variables selected were percent of drug release at 2nd hour (*Y*_1_) and at 8th hour (*Y*_2_). Response surface plots were developed, and optimum formulations were selected on the basis of desirability. The formulated tablets showed anomalous release mechanism and followed matrix drug release kinetics, resulting in regulated and complete release from the tablets within 8 to 10 hours. The polymer carboxymethyl xyloglucan and HPMC K100M had significant effect on drug release from the tablet (*P* > 0.05). Polynomial mathematical models, generated for various response variables using multiple regression analysis, were found to be statistically significant (*P* > 0.05). The statistical models developed for optimization were found to be valid.

## 1. Introduction

Hydrophilic matrices are an interesting option while developing an oral sustained-release formulation. They can be used for controlled release of both water-soluble and water-insoluble drugs. The release behaviour of drugs varies with the nature of the matrix and it is the complex interaction of swelling, diffusion, and erosion processes [1]. Polysaccharides are the choice of material which has been evaluated as hydrophilic matrix for drug delivery system due to their nontoxicity and acceptance by regulating authorities.

Xyloglucan is a natural polysaccharide isolated from seed kernel of *Tamarindus indica*. It is used as ingredient in food and pharmaceutical industry. It has been significantly evaluated for use in hydrophilic drug delivery system. It possesses high viscosity, broad pH tolerance, and swelling and binding properties [2]. This led to its application as release retardant polymer and binder in pharmaceutical industry. In addition to these, other important properties of xyloglucan have been identified recently, which include noncarcinogenicity [3], mucoadhesivity, biocompatibility [4], high drug holding capacity [5], and high thermal stability [6]. This led to its application as excipient in hydrophilic drug delivery system [3–6].

Carboxymethyl xyloglucan is a derivative of xyloglucan and the microbial resistance of CM-xyloglucan is much better than that of plain powder. The viscosity of CM-xyloglucan in solutions is higher compared to native gum. Derivatization of xyloglucan, that is, CM-xyloglucan, disrupts the organization and exposes the polysaccharide network for hydration which results in higher viscosity and due to this its swelling index is also higher as compared to Xyloglucan. The presence of carboxymethyl groups makes the molecule resistant toward enzymatic attack [7]. Since carboxymethyl xyloglucan is having improved properties which are required for the retardation of release, the present study was undertaken to elucidate release kinetics of water-soluble drug from the matrix.

Tramadol, a synthetic opioid of the aminocyclohexanol group, is a centrally acting analgesic with weak opioid agonist properties. The half-life of the drug is about 5.5 hours and the usual oral dosage regimen is 50 to 100 mg every 4 to 6 hours with a maximum dosage of 400 mg/day [8]. To reduce the frequency of administration and to improve patient compliance, a sustained-release formulation of tramadol is desirable. The drug is freely soluble in water and hence judicious selection of release retarding excipients is necessary to achieve a constant *in vivo* input rate of the drug. The most commonly used method of modulating the drug release is to include it in a matrix system [9]. The major objective of the present investigation was to develop a sustained-release drug delivery system using simplex centroid design as an optimization technique.

## 2. Material and Methods

Carboxymethyl xyloglucan (CM-xyloglucan) was procured from Encore Natural Polymer Private Limited, Ahmedabad. HPMC (K100 M), dicalcium phosphate were purchased from SD Fine Chemicals Ltd (Mumbai, India). PVP K-30 was procured from Loba Chemicals (Mumbai, India). Tramadol HCl was a gift sample from Rantus Pharma Ltd (Hyderabad). All the other chemicals used were of high analytical grade. 

### 2.1. Methods

#### 2.1.1. Preparation of Matrix Tablets

**Figure 1:**
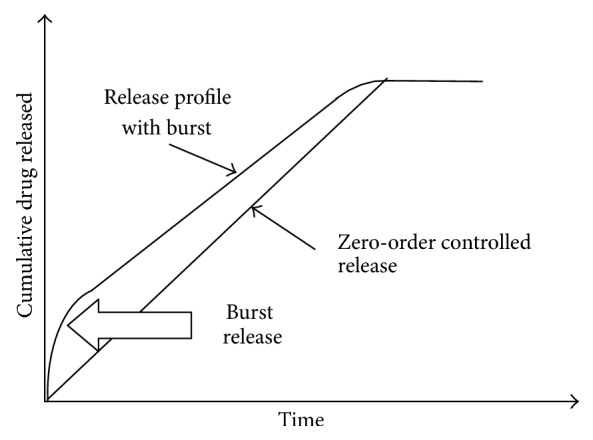
Diagram indicating the burst effect phenomenon.

Matrix tablets, each containing 100 mg of Tramadol HCl, were prepared. For determining levels of carboxymethyl xyloglucan, initial trial batches with different concentrations of carboxymethyl xyloglucan were prepared and evaluated for physico-chemical properties of formulation and dissolution studies. In the trial runs, carboxymethyl xyloglucan concentration was varied from 50 to 250 mg. It was observed that as the concentration of carboxymethyl xyloglucan increased, the retarding effect of the formulation also increased, but a phenomenon of burst effect was prominently seen in all the formulations (Figure 1). Hence, to prevent the burst effect HPMC K100M was used. The quantities of other ingredients were kept constant, that is, DCP at 20 mg. Magnesium stearate and talc at 5 mg were used as a lubricant and a glidant, respectively. 

Different tablet formulations were prepared by wet granulation technique. All the powders were passed through a sieve of 80 mesh size. Required quantities of drug, polymer, and dicalcium phosphate were mixed thoroughly and a sufficient volume of granulating agent (isopropyl alcohol solution of PVP K-30) was added slowly. After enough cohesiveness was obtained, the mass was sieved through 22/44 mesh. The granules were dried at room temperature. Once dry, the granules retained on 44 mesh were mixed with 15% of fines (granules that passed through 44 mesh). Talc and magnesium stearate were finally added as glidant and lubricant, respectively. The tablets were compressed (10 mm diameter, flat punches) using a tablet compression machine, Mini Press-II MT, Rimek. Each tablet contained 100 mg of tramadol HCl and other pharmaceutical ingredients. Prior to the compression, the granules were evaluated for several tests.

#### 2.1.2. Evaluation of Tablets

The tablets were evaluated for different physicochemical parameters such as angle of repose [10], thickness, bulk density, tap density [11], Carr's index [12], weight variation, thickness, hardness, friability, weight variation [13], drug content, and *in vitro *release. In drug content, 20 tablets of each formulation were weighed and powdered. The quantity of powder equivalent to 100 mg of tramadol HCl was taken and dissolved in 30 mL of distilled water; sufficient amount distilled water was added to produce 50 mL and was filtered. The absorbance was measured spectrophotometrically at 271 nm after suitable dilution. The *in vitro *dissolution studies were carried out using USP apparatus type II (DA 6D, Veego) at 100 rpm. The dissolution medium consisted of 0.1 N hydrochloric acid for the first 2 hours and the phosphate buffer pH 6.8 from 3 to 8 hours (900 mL), maintained at 37°C ± 0.5°C. The drug release at different time intervals was measured by UV-visible spectrophotometer (V-530, Jasco) at 271 nm. The release studies were conducted in triplicate (6 tablets in each set) and the mean values were plotted versus time with standard deviation (SD) of less than 3, indicating the reproducibility of the results.

*Kinetic Modeling* of *Drug Release*. The dissolution profile of all the batches was fitted to various models such as zero-order, first-order [14], MatrixT [15], Hixon-Crowell [16], Korsmeyer et al. and Peppas [17, 18], and Weibull models to ascertain the kinetic modeling of drug release. The method of Bamba et al. [19] was adopted for deciding the most appropriate model.

*Swelling Index*. The extent of swelling was measured in terms of percent weight gain by the tablet [20]. The swelling behavior of all formulations was studied. One tablet from each formulation was kept in separate Petri dishes containing 0.1 N HCl for first 2 hours and then was placed in phosphate buffer pH 6.8 till 8 hours. At the end of every 1 hour interval, the tablet was withdrawn, soaked with tissue paper, and weighed. This process was continued till the end of 8 hours. Percentage weight gain by the tablets was calculated by formula

(1) $$\begin{array}{c} \text{SI}=\left\{\frac{\left(M_{t}-M_{0}\right)}{M_{0}}\right\}\times 100, \end{array}$$

where, SI is the swelling index, *M*_*t*_ is the weight of swollen tablets at respective time intervals, and *M*_0_ is the weight of tablet at time (*t*) = 0.

*Similarity Factor*. The similarity factor (*f*) is used for release profile comparison between the test formulation and marketed preparation [21]. It is a logarithmic transformation of the sum squared error of differences between the test *T*_*j*_ and reference products *R*_*j*_ over all time points:

(2) $$\begin{array}{c} f_{2}=50\times \log\left\{{\left[1+\left(\frac{1}{n}\right)\sum_{j=1}^{n}{\left|R_{j}-T_{j}\right|}^{2}\right]}^{-0.5}\times 100\right\}, \end{array}$$

where “*n*” is number of pull points, “*R*_*j*_” is reference profile at time point *t*, and “*T*_*j*_” is test profile at same time point *t*. The similarity factor fits the result between 0 and 100 as shown in Table 11. It is 100 when test and reference profile are identical and tends to 0 when dissimilarity increases.

**Figure 2:**
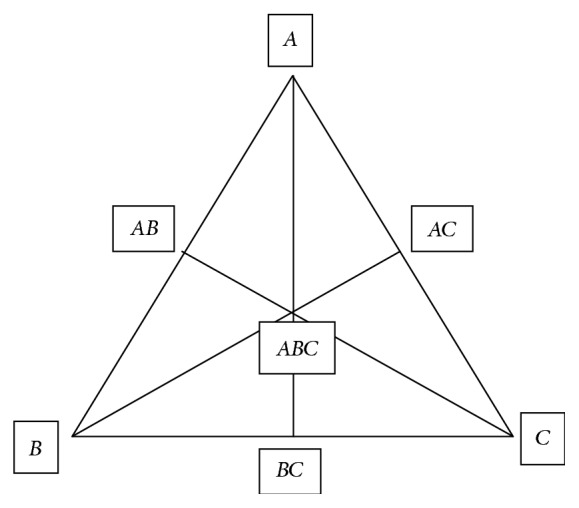
Equilateral triangle representing simplex lattice design for 3 components (*A*, *B*, and *C*).

*Simplex Centroid Design. *A simplex centroid design [22] was adopted to optimize the formulation variables. In this design three factors were evaluated by changing their concentrations simultaneously and keeping their total concentration constant. The simplex centroid design for a 3-component system (*A*, *B*, and *C*) is represented by an equilateral triangle in a 2-dimensional space (Figure 2). The amounts of matrixing agent carboxymethyl xyloglucan (carboxymethyl xyloglucan, *X*_1_), gelling agent (HPMC K100M, *X*_2_), and dicalcium phosphate (DCP, *X*_3_) were selected as independent variables as shown in Table 3. Percent release values of drug at 2nd hour and 8th hour were selected as dependent variables. The levels of the three factors were selected on the basis of the preliminary studies carried out before implementing the experimental design. 

### 2.2. Statistical Analysis

The statistical analysis of the simplex centroid design batches was done using Design-Expert 8.0.5 software. To study the influence of each factor on response and behavior of the system within the designed space, response surface plots were generated.

## 3. Result and Discussion

### 3.1. Preliminary Trials

In trial batches from *A*_1_ to *A*_5_ CM-xyloglucan was used in the range of 50 to 250 mg (Table 1); it displayed a retardation of drug release commensurate to the concentration of polymer.

For the comparison of native xyloglucan and derivative carboxymethyl xyloglucan, trial batches *M*_1_ and *M*_2_ each containing 150 mg polymer were prepared (Table 12). Batch *M*_1_ containing plain xyloglucan sustained-release only for 4 hours while batch *M*_2_ containing CM-xyloglucan sustained-release up to 7 hours (Figure 10). 

**Figure 3:**
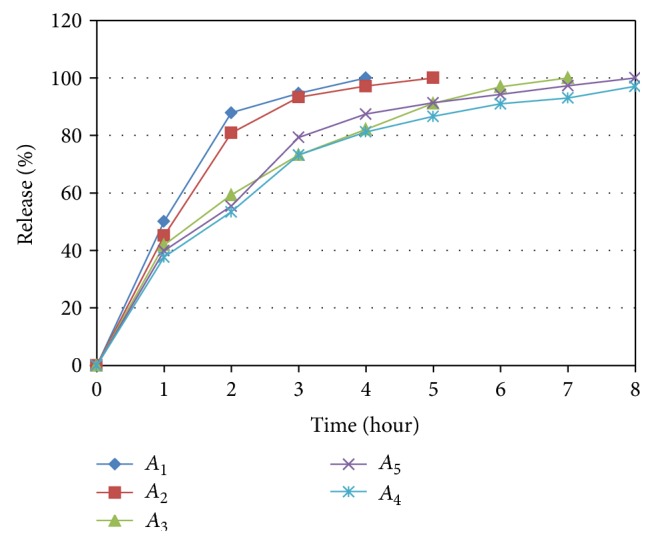
Dissolution profile for batches *A*_1_ to *A*_5_.

The percentage drug release at second hour for *A*_1_ to *A*_5_ formulations was in the range of 87.90% to 53.39%, respectively, as shown in Figure 3, while only *A*_3_ to *A*_5_ formulations were able to retard drug release up to 7 to 8 hours. It was found from the initial trials that 150 to 250 mg of polymer is required for sustaining drug release up to 8 hours, while only *A*_3_ to *A*_5_ formulations were able to retard drug release up to 7 to 8 hours. But at 150 mg concentration (*A*_3_ batch) release was sustained up to 7 hours so that minimum level should be slightly more than 150 mg, while for 200 and 250 mg concentration release was almost similar, hence, 200 mg was decided as a higher level of the polymer. 

The granule characterization of the trial batch formulations (*A*_1_ to *A*_5_) was performed. The results of this study are depicted in Table 2 which shows excellent flow properties and compressibility. 

### 3.2. Swelling Study

**Figure 4:**
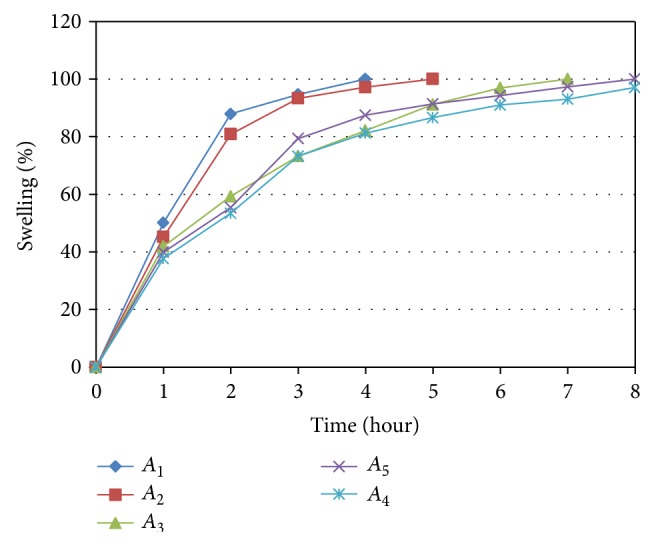
Swelling index for *A*_1_ to *A*_5_ formulations.

Swelling study results showed slow and gradual increase in swelling index with time and concentration of CM-xyloglucan, as CM-xyloglucan does not swell instantaneously as soon as it comes in contact with water as shown in Figure 4; this results in burst release due to improper swelling. Therefore, to avoid the burst release effect, we have added HPMC K100 in the small quantity. Swelling of *A*_5_ batch is highest because it contains a higher amount of polymer. If swelling is more then it increases the path length required for water to travel inside the core of tablet, which gives more sustained-release. 

### 3.3. Optimization of the Release Rate of Tramadol HCl

The simplex mixture designs are useful when the performance of formulation depends upon relative proportion of ingredients and not on the concentration. The amount of ingredients can be varied keeping the total concentration constant. This design is useful in formulation situations.

Simplex centroid mixture design is selected for optimization of excipient levels when there are more than two variables. In this design, all the factors are studied in all the possible combinations, as it is considered to be most efficient in estimating the influence of individual variables (main effects) and their interactions, using minimum experimentation. The amounts of matrixing agent CM-xyloglucan (*X*_1_), gelling agent (HPMC K100M, *X*_2_), and dicalcium phosphate (DCP, *X*_3_) were selected as independent variables. Percent release values of drug at second hour and eighth hour were selected as dependent variables. Dissolution profile of USFDA approved tramadol HCl twice a day. SR tablets gave the following limit for percent drug release 1 hr (20–50), 2 hr (40–75), 4 hr (60–95), 8 hr (80–100), and 12 hr (90–100) [23]. To optimize the formulation for acceptance criteria, that is, percent drug release at second hour (rel_2_ Hr) should be in between 20 to 50 and percent drug release at eighth hour (rel_8_ Hr) should be in between 80 to 100, a simplex centroid design was used. The levels of the three factors were selected on the basis of the preliminary studies carried out before implementing the experimental design. 7 formulations (*F*_1_–*F*_7_) were prepared as per the experimental design and evaluated for chosen response variables (Tables 4 and 6).

**Figure 5:**
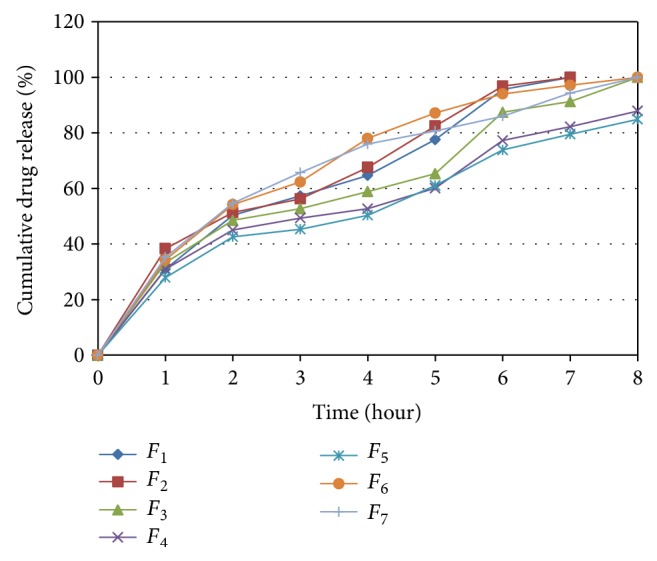
Dissolution profile for batches *F*_1_ to *F*_7_.

Hence, it was decided to optimize the amount of CM-xyloglucan between 180 to 200 mg per tablet in order to have a sustained and complete release of drug at the end of eight to twelve hours. The amounts of matrixing agent carboxymethyl xyloglucan (CM-xyloglucan, *X*_1_), gelling agent (HPMC K100M, *X*_2_), and dicalcium phosphate (DCP, *X*_3_) were selected as independent variables. A statistical model incorporating 7 interactive terms was used to evaluate the responses:

(3) Y=b0+b1X1+b2X2+b3X3+b8X1X2+b23X2X3+  b13X1X3+b83X1X2X3,

where *Y* is the dependent variable, *b*_0_ is the arithmetic mean response of the 7 runs, and *b*_*i*_ is the estimated coefficient for the factor *X*_*i*_. The main effects (*X*_1_, *X*_2_, and *X*_3_) represent the average result of changing one factor at a time from its low to high value. The interaction terms (*X*_1_ *X*_2_, *X*_2_ *X*_3_, *X*_1_ *X*_3_, and *X*_1_ *X*_2_ *X*_3_) show how the response changes when two or more factors are simultaneously changed. The rel_2_ Hr and rel_8_ Hr, respectively, for all 7 batches (*F*_1_–*F*_7_) showed a wide variation (Figure 5). The data clearly indicate that the values of drug release are strongly dependent on the selected independent variables. 

**Figure 6:**
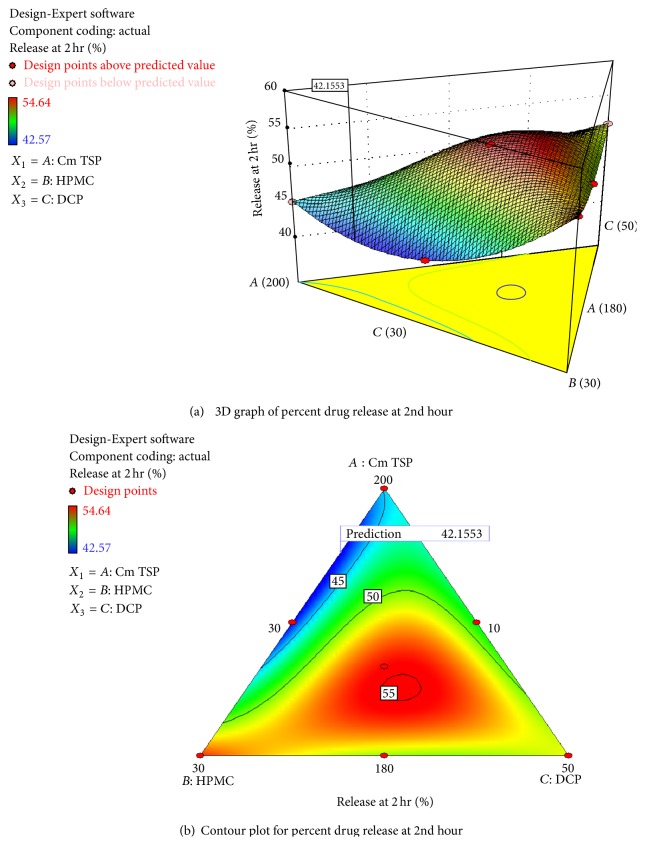
Contour plot showing amount of drug release at second hour (*Y*_1_) using different combinatinon, of *X*_1_, *X*_2_, and *X*_3_. The contour lines showing percentage of drug release at the end of second hour.

**Figure 7:**
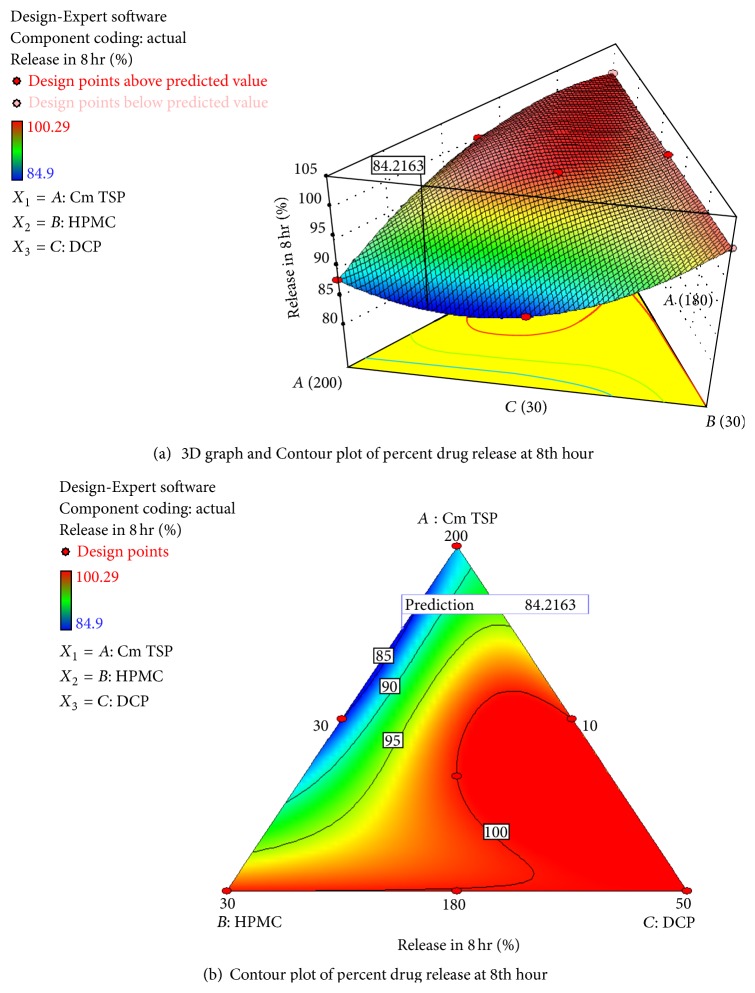
Contour plot showking amount of drug release at twelfth hour (*Y*_2_) using different combinatinon of *X*_1_, *X*_2_, and *X*_3_. The contour lines showing percentage of drug release at the end of twelfth hour.

To demonstrate graphically the effect of release modifying polymers on the dissolution profile, contour plots and 3D graphs were generated. The 3D graph (as shown in Figure 6(a)) for rel_2_ Hr shows that percent drug release at second hour is plotted on *y*-axis where as the concentrations of excipients were plotted on *x*- and *z*-axis. As the concentration of carboxymethyl xyloglucan and HPMC K100M increased from 180 to 200 mg and 10 to 30 mg, respectively, the percent drug release decreased signifying that the polymers have definite effect on drug release, and especially along the axis region of HPMC-K-100M the effect was greater at lower concentration which indicate its effectiveness in controlling burst release is prominent at lower level, whereas the increasing concentration of DCP shows significant effect on drug release at lowest concentration. The contour plot (as shown in Figure 6(b)) for rel_2 _ Hr justifies that optimum formulation complying with the acceptance criteria can be achieved by selecting the formulations near to the upper left side of the triangle-shaped contour plot which is the diagram obtained from the evaluation result of (*F*_1_–*F*_7_) formulations. Almost similar results were observed with 3D graph (as shown in Figure 7(a)) and contour plot (as shown in Figure 7(b)) for rel_8_ Hr. Here as the concentration of carboxymethyl xyloglucan increased release retardation effect, also increases due to increase in the diffusion path length and DCP is showing its effect at lower concentration but not at higher concentration, while HPMC-K-100 M is helping to control burst release in initial hours but not showing any release retarding effect at eight hour because it is used in a smaller quantity. 

From the dissolution study of seven batches (Figure 5), percent release of drug at two hours (rel_2_ Hr) was found to be in range of 27.93% to 38.33% and percent release of drug at eight hour (rel_8_ Hr) was found to be in range of 84.90% to 100.21%.

#### 3.3.1. Percent Drug Release at Second Hour (rel_2_ Hr)

Statistical optimization was carried out in Design-Expert software (version 8.0.5), which suggested that special cubic model (SCM) was followed for release at two hour that with *P* value of 0.0409. This indicated that the model was significant. Therefore, SCM was selected for percent release at two hours (rel_2_ Hr). In order to find out the contribution of each components and their interaction, analysis of variance (ANOVA) for SCM was carried.  Table 10 shows the results of the analysis of variance (ANOVA), which was used to generate mathematical models. The model *F*-value (344.65) implied that the model was significant. Value of probability (*P*) less than 0.05 indicates that model terms were significant. In this case, linear mixture components, *AB*, *BC*, and *ABC*, were significant model terms 

 The equation for percent drug release at the end of the second hour is

(4) Y1=+45.15∗A+54.19∗B+51.44∗C−28.40∗A∗B−9.78∗B∗C+232.81∗A∗B∗C,

where, *A* is the carboxymethyl xyloglucan, *B* is the HPMC, *C* is the DCP, *Y*_1_ is the % release at second hour.

#### 3.3.2. Percent Drug Release at Eighth Hour (rel_8_ Hr)

Model adequacy was checked for percent drug release at eighth hour (rel_8_ Hr). Model gave the highest order polynomial where the additional terms were significant and the model was selected. The SCM suggested by the software followed for (rel_8_ Hr), with *P*-value 0.0004 and this indicated the model was highly significant. In order to find out contribution of each component and their interaction, ANOVA for SCM was carried out. The model *F*-value of 326887 implied that the model was highly significant. Value of *P* less than 0.05 indicates that model terms were significant. In this case, linear mixture components *AB*, *AC*, and *A* ^2^ *BC* were significant model terms. The equation for percent drug release at the end of eighth hour is

(5) Y2=+87.89∗A+100.00∗B+100.29∗C−36.18∗A∗B+23.64∗A∗C+431.97∗A2∗B∗C,

where *A*: carboxymethyl xyloglucan, *B*: HPMC, and *C*: DCP, *Y*_2_: % release at eighth hour.

#### 3.3.3. Search for Optimum Formulation

Based on acceptance criteria and desirability factor, Design-Expert suggested three optimum formulations *B*_1_, *B*_2_, and *B*_3_ (as shown in Table 7). The values of desirability closest to 1 is considered most favourable. The values of desirabilities for three optimized formulations *B*_1_, *B*_2_, and *B*_3_ were 0.999, 0.987, 0.786, respectively. Optimized batches were formulated with the suggested composition. The physicochemical evaluation (as shown in Table 8) of optimum formulations showed good flow properties and excellent compression characteristics. 

### 3.4. Validation of Optimized Model

**Figure 8:**
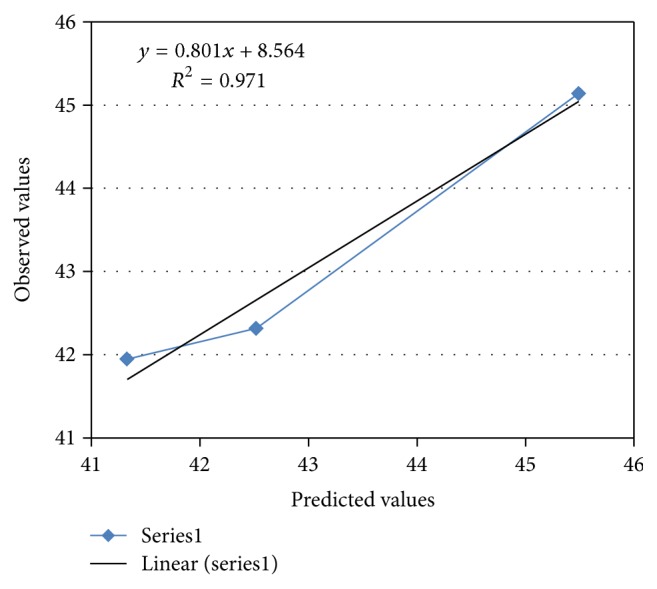
Linear correlation plot for release at 2nd hour.

**Figure 9:**
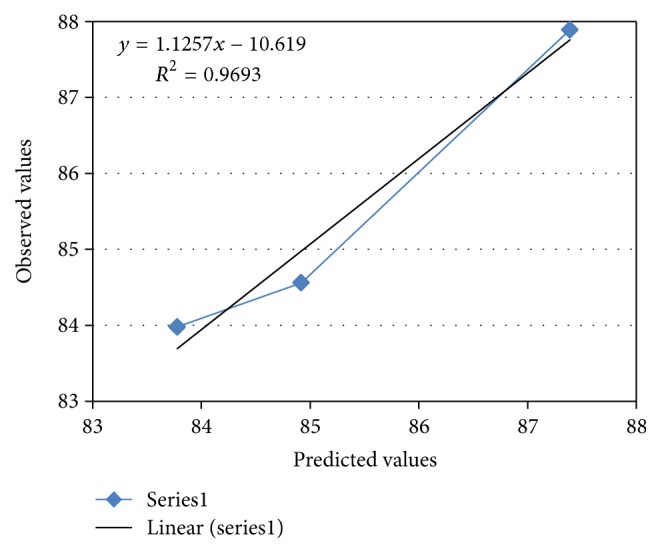
Linear correlation plot for release at 8th hour.

**Figure 10:**
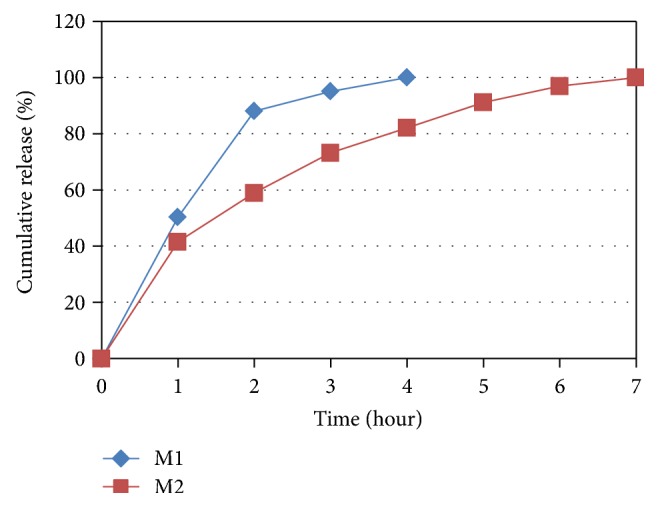
Dissolution profile for batches *M*_1_ to *M*_2_.

For all three optimized formulations, the results of the physical evaluations and tablet assay were found to be within limits. Figures 8 and 9 show linear correlation plots drawn between the predicted and observed response variables. These plots demonstrated higher values of *R* as 0.971 and 0.969 for release at 2nd hour and release at 8th hour, respectively, indicating a good correlation. Upon comparison of the observed responses with that of the anticipated responses, the prediction error varied between −1.44% and +0.775% as shown in Table 9. Thus, the low magnitudes of error as well as the significant values of *R* ^2^ in the current study indicate a high prognostic ability of the software.

The optimization batches were subjected to short-term stability studies at 40°C and 75% relative humidity (RH) for 3 months. Samples withdrawn after 3 months showed no significant change in dissolution studies and drug content.

## 4. Conclusion

Sustained drug release following matrix kinetics attained in the current study indicates that the hydrophilic matrix tablet prepared using carboxymethyl xyloglucan and HPMC-K-100M can successfully be employed sustain the drug release up to 8 to 12 hours as shown in Table 5. carboxymethyl xyloglucan played major role in sustaining release of tramadol at later stage of release profile, whereas HPMC-K-100M prevented the burst effect by controlling the sudden release of drug from the dosage form at the initial stage of the release profile. It was concluded that appropriate balancing between various levels of the polymers may contribute better results. High degree of prognosis obtained using RSM corroborates that a simplex centroid design is quite efficient in optimizing drug delivery systems.

## Figures and Tables

**Table 1:** Trial batch formulations from *A*_1_ to *A*_5_.

Ingredients	Formulations				
	*A*_1_	*A*_2_	*A*_3_	*A*_4_	*A*_5_
Tramadol HCl	100	100	100	100	100
TSP	50	100	150	200	250
HPMC K100M	—	—	—	—	—
DCP	20	20	20	20	20
Talc	5	5	5	5	5
Magnesium Stearate	5	5	5	5	5
PVP K-30	30	30	30	30	30
Isopropyl alcohol	qs.	qs.	qs.	qs.	qs.

Total	210	260	310	360	410

All quantities in mg.

**Table 2:** Dissolution model for formulations from *A*_1_ to *A*_5_.

Batch code	Release exponent (*n*)	Kinetic constant (*K*)	*R *	Best fit model
*A*_1_	0.6948	32.71	0.9692	Matrix
*A*_2_	0.7634	28.67	0.9736	Matrix
*A*_3_	0.7791	25.36	0.9795	Matrix
*A*_4_	0.8372	19.82	0.9817	Matrix
*A*_5_	0.6834	15.26	0.9854	Matrix

Non-Fickian.

**Table 3:** Coded levels and their corresponding actual values of ingredients.

Coded	Actual values		
level	Carboxymethyl xyloglucan	HPMC (K100M)	DCP

0	180	10	30
0.33	186.67	16.67	36.67
0.5	190	20	40
1	200	30	50

All quantities in mg.

**Table 4:** Composition of *F*_1_ to *F*_7_ formulations.

Ingredients	Formulations (mg)						
	*F*_1_	*F*_2_	*F*_3_	*F*_4_	*F*_5_	*F*_6_	*F*_7_

Tramadol HCl	100	100	100	100	100	100	100
Carboxymethyl xyloglucan	200	180	180	190	190	180	186.67
HPMC-k-100M	10	30	10	20	10	20	16.67
DCP	30	30	50	30	40	40	36.67
Talc	5	5	5	5	5	5	5
Magnesium stearate	5	5	5	5	5	5	5
PVP K-30	30	30	30	30	30	30	30
Isopropyl alcohol	qs.	qs.	qs.	qs.	qs.	qs.	qs.

Total	380	380	380	380	380	380	380

All quantities in mg.

**Table 5:** Dissolution model for *F*_1_ to *F*_7_ formulations.

Batch code	Release exponent (*n*)	Kinetic constant (*K*)	*R *	Best fit model
*F*_1_	0.5919	30.8556	0.9874	Matrix
*F*_2_	0.5030	36.2863	0.9828	Matrix
*F*_3_	0.5171	31.8826	0.9845	Matrix
*F*_4_	0.5008	29.6438	0.9870	Matrix
*F*_5_	0.5301	27.1364	0.9897	Matrix
*F*_6_	0.5814	36.9647	0.9955	Matrix
*F*_7_	0.5049	36.9443	0.9957	Matrix

Non-Fickian.

**Table 6:** Pre- and postcompression properties of optimization batches *F*_1_–*F*_7_.

Parameters	*F*_1_	*F*_2_	*F*_3_	*F*_4_	*F*_5_	*F*_6_	*F*_7_
Bulk density (g/mL)	0.387 ± 0.22	0.355 ± 0.17	0.303 ± 0.11	0.409 ± 0.30	0.343 ± 0.11	0.318 ± 0.16	0.366 ± 0.15
Tapped density (g/mL)	0.442 ± 0.14	0.416 ± 0.35	0.426 ± 0.03	0.493 ± 0.26	0.428 ± 0.05	0.374 ± 0.04	0.412 ± 0.04
Angle of repose	27.78 ± 0.14	26.05 ± 0.08	25.55 ± 0.12	29.85 ± 0.02	28.08 ± 0.16	27.08 ± 0.08	26.06 ± 0.04
Carr's index	13.44 ± 0.14	12.66 ± 0.11	12.3 ± 0.16	14.43 ± 0.13	14.06 ± 0.12	13.97 ± 0.17	12.17 ± 0.15
Hausner's ratio	1.14 ± 0.18	1.17 ± 0.28	1.41 ± 0.07	1.21 ± 0.28	1.24 ± 0.08	1.17 ± 0.13	1.13 ± 0.11
Hardness (kg/cm^2^)	5.1 ± 0.33	5.3 ± 0.26	5.5 ± 0.18	5.4 ± 0.34	5.7 ± 0.17	5.5 ± 0.32	5.4 ± 0.13
Friability	0.74 ± 0.21	0.70 ± 0.11	0.57 ± 0.22	0.62 ± 0.16	0.42 ± 0.12	0.52 ± 0.23	0.67 ± 0.22
Uniformity of weight (mg)	378.29 ± 0.34	379.78 ± 0.45	378.98 ± 0.55	381.19 ± 0.36	382.4 ± 0.12	378.56 ± 0.24	381.78 ± 0.13
Drug content (%)	100.22 ± 0.22	100.62 ± 0.11	99.14 ± 0.18	98.34 ± 0.39	98.72 ± 0.21	101.16 ± 0.67	98.52 ± 0.89
Thickness (mm)	4.40 ± 0.014	4.17 ± 0.016	3.89 ± 0.026	4.29 ± 0.016	4.02 ± 0.031	4.22 ± 0.014	3.83 ± 0.034

±SD (standard deviation) *n* = 3.

**Table 7:** Composition of optimum formulations *B*_1_, *B*_2_, and *B*_3_.

Composition (mg)	Formulations		
	*B*_1_	*B*_2_	*B*_3_

Tramadol HCl	100	100	100
Carboxymethyl xyloglucan	194.39	190.606	200
HPMC-K-100M	15.610	19.394	10
DCP	30	30	30
Talc	5	5	5
Magnesium stearate	5	5	5
PVP K-30	30	30	30
Isopropyl alcohol	qs.	qs.	qs.

**Table 8:** Pre- and postcompression properties of optimized carboxymethyl xyloglucan matrix tablets.

Parameters	*B*_1_	*B*_2_	*B*_3_

Bulk density	0.312 ± 0.19	0.331 ± 0.22	0.376 ± 0.16
Tapped density	0.351 ± 0.24	0.348 ± 0.13	0.397 ± 0.09
Angle repose	25.03 ± 0.07	27.31 ± 0.11	29.27 ± 0.03
Carr's index	13.01 ± 0.20	13.22 ± 0.17	14.21 ± 0.13
Hausner's ratio	1.12 ± 0.21	1.05 ± 0.19	1.06 ± 0.14
Hardness	5.2 ± 0.03	5.1 ± 0.01	5.3 ± 0.04
Friability	0.542 ± 0.07	0.481 ± 0.16	0.768 ± 0.18
Weight variation	365 ± 1.79	387 ± 0.93	395 ± 1.04
Drug content	99.69 ± 0.17	98.61 ± 0.02	98.14 ± 0.11
Thickness (mm)	3.82 ± 0.03	3.96 ± 0.06	4.17 ± 0.01

±SD (standard deviation) *n* = 3.

**Table 9:** The predicted and experimental values of response variables, and their percentage prediction error for *B*_1_, *B*_2_, and *B*_3_ formulations.

Optimized formulation	Response variable	Experimental value	Predicted value	Percentage prediction error

*B*_1_	*Y*_1_	41.33%	41.95%	−1.44
	*Y*_2_	83.78%	83.98%	−0.238

*B*_2_	*Y*_1_	42.52%	42.32%	+0.47
	*Y*_2_	84.92%	84.56%	+0.425

*B*_3_	*Y*_1_	45.49%	45.14%	+0.775
	*Y*_2_	87.39%	87.89%	−0.568

**Table 10:** Analysis of variance (ANOVA) for all two responses.

	% Release at 2 hour			% Release at 8 hour	
Source			Source		
	*F*-value	*P* value		*F*-value	*P* value
Model	344.65	0.0409	Model	272.39	0.004
Linear mixture	389.03	0.0358	Linear mixture	162.92	0.0003
*AB *	490.78	0.0287	*AB *	56.11	0.0003
*BC *	58.17	0.0830	*AC *	23.94	0.0005
*ABC *	741.53	0.0234	*A* ^2^ *BC*	19.97	0.0006

**Table 11:** Similarity factor determination.

Optimized formulations	*f*_2_ value	Consideration
*B*_1_	75	Similar
*B*_2_	70	Similar
*B*_3_	61	Similar

**Table 12:** Trial batch formulations of plain xyloglucan and carboxymethyl xyloglucan.

Ingredients	Formulations	
	*M*_1_	*M*_2_

Tramadol HCl	100	100
TSP	150	—
CM-TSP	—	150
DCP	20	20
Talc	5	5
Magnesium stearate	5	5
PVP K-30	30	30
Isopropyl alcohol	qs.	qs.

Total	310	310
